# The use of Modafinil on Methamphetamine withdrawal in mechanically ventilated patients

**DOI:** 10.1016/j.rmcr.2025.102248

**Published:** 2025-06-30

**Authors:** Kristina Akopyan, Anastasia Engeleit, Minh Nguyen, Adam Austin

**Affiliations:** a Division of Hospital Medicine, Department of Medicine, University of Florida, Gainesville, FL, USA; b Medical Intensive Care Unit, Department of Pharmacy, UF Health Shands Hospital, Gainesville, FL, USA; c Department of Medicine, Division of Pulmonary, Critical Care and Sleep Medicine, University of Florida, Gainesville, FL, USA

**Keywords:** Modafinil, Methamphetamine, Mechanical ventilation, Respiratory failure

## Abstract

Modafinil has been used to treat methamphetamine dependence, and the medication's stimulant properties have also been shown to alleviate methamphetamine withdrawal symptoms. We present two cases of patients with history of methamphetamine use who required intubation and mechanical ventilation, one in the setting of opiate withdrawal, and the other with severe asthma exacerbation causing cardiac arrest. Both patients remained somnolent after intubation and mechanical ventilation. Given suspected methamphetamine withdrawal, they both received modafinil and wakefulness greatly improved allowing them to be extubated. These cases demonstrate that there needs to be further investigation into the use of modafinil for methamphetamine withdrawal, especially in the setting of mechanical ventilation.

## 1 Introduction

Methamphetamine is a synthetic stimulant that is approved for the treatment of attention deficit hyperactivity disorder (ADHD), but there are 1.2 million Americans who use methamphetamine illicitly at least once per year. Modafinil is a non-amphetamine psychostimulant that has been approved for treatment of narcolepsy, shift work sleep disorder, and as an adjunct for obstructive sleep apnea (OSA). Modafinil has also been used to treat methamphetamine dependence, and its stimulant properties have been shown to alleviate methamphetamine withdrawal symptoms [1]. Modafinil increases levels of dopamine, norepinephrine, serotonin, glutamate, and histamine while decreasing levels of γ-aminobutyric acid (GABA) [2]. Modafinil can increase alertness by stimulating the brain, and it can also stimulate breathing rate such as in narcolepsy and OSA [3]. To the best of our knowledge, the use of modafinil in mechanically ventilated patients has not been reported. We report the case of a 43-year-old female with history of bipolar disorder, generalized anxiety disorder (GAD), post-traumatic stress disorder (PTSD), and substance abuse disorder including methamphetamine use who was intubated for airway protection in the setting of opiate withdrawal. She has prolonged somnolence on mechanical ventilation that improves after the use of modafinil. The second case we report is a 39-year-old male with history of severe persistent asthma and methamphetamine use who is intubated after cardiac arrest secondary to a severe asthma exacerbation. He also has prolonged somnolence on mechanical ventilation which improves after a single dose of modafinil allowing him to be extubated. These cases emphasize the need for further research into the use of modafinil for mechanically ventilated patients with a history of methamphetamine use (see Table 1).

**Table 1:** Summary of cases.

Cases	Age	Sex	Urine Drug Screen	Sedation	First intubation days	Second intubation days	Dose of Modafinil
1	43	Female	Amphetamine, cannabinoids, fentanyl	Propofol, Fentanyl	3	8	200 mg
2	39	Male	Amphetamine	Propofol, Fentanyl, Ketamine	6	2	50 mg

Clinical course including urine drug screen, sedation, number of intubation days, and dose of modafinil given.

## 2 Case 1

A 43-year-old female with history of bipolar disorder, GAD, PTSD, and substance abuse disorder was found on the floor with dry vomit on her face and nearby drug paraphernalia. She was brought to the emergency department by emergency medical services. She was manic and making comments of suicidal ideation. Her home medications included aripiprazole, escitalopram, and trazodone. Her urine drug screen (UDS) was positive for amphetamines, cannabinoids, and fentanyl. CPK was noted to be greater than 30,000 U/L. She received 2 doses of naloxone and then became tachypneic and hypertensive, requiring intubation for airway protection. She was sedated with propofol and fentanyl infusion. She was found to have aspiration pneumonia and treated with ceftriaxone. She received aggressive fluid resuscitation with lactated ringers at 200 mL/hr for rhabdomyolysis. After 3 days of spontaneous breathing trials (SBTs), she was subsequently extubated. She started to have recurrent fevers and changes to mental status. She was also found to have hypertonicity in her extremities and proximal greater than distal weakness. She had evidence for infection with WBC count of 44.6 k/uL, CRP of 243 mg/L, and procalcitonin of 2.20 ng/mL. CT and MRI brain showed findings of anoxic brain injury to the basal ganglia and corona radiata. Neurology was consulted and recommended a lumbar puncture. This was unable to be obtained due to the patient's inability to follow commands. She was started on broad-spectrum antibiotics including ampicillin, acyclovir, piperacillin-tazobactam, and vancomycin. Interventional radiology was consulted, and lumbar puncture was obtained with sedation. CSF studies showed no signs of infection. Acyclovir and ampicillin were discontinued. She continued to have altered mentation and agitation 5 days after extubation. Due to her vomiting and inability to protect her airway, she was intubated again. She remained somnolent and non-responsive while on mechanical ventilation. EEG showed mild generalized slowing with brief periods of generalized rhythmic delta activity suggestive of mild diffuse cerebral dysfunction. There were no electrographic seizures or epileptiform discharges seen. Her anoxic brain injury was thought to be post-anoxic parkinsonism. Psychiatry was also consulted due to suspected neuroleptic malignant syndrome (NMS) due to hyperthermia and elevated CK. She was started on bromocriptine and then transitioned to carbidopa-levodopa. Malignant catatonia was also considered, but she did not respond to lorazepam challenge. Her somnolence continued for the next 5 days after re-intubation despite remaining off sedation for 3 days. She was given modafinil 200 mg and then started to become more awake. She was given the medication once daily for the next 3 days. Her Richmond-Agitation Sedation Scale (RASS) score was noted to change from −4 (deep sedation) to 0 (alert and calm). She was then extubated. She continued to receive modafinil 200 mg daily for the next 10 days while she was hospitalized. She was discharged with modafinil 200 mg daily due to her improved mentation while taking it.

## 3 Case 2

39-year-old male with history of severe persistent asthma, four prior intubations for asthma exacerbations, and substance abuse including cocaine and methamphetamine had cardiac arrest at home with cardiopulmonary resuscitation (CPR) started by family. Emergency medical services arrived and intubated him on the scene. They continued CPR, and there was return of spontaneous circulation (ROSC) after a total of 15 minutes of CPR. He received continuous albuterol, terbutaline, and epinephrine infusion for bronchospasm. UDS was positive for methamphetamines. CT chest w/o contrast showed small bi-apical pneumothoraxes, small pneumo-mediastinum, scattered tree-in-bud nodules, and acute buckle fractures of the anterior right fourth to sixth ribs. He required placement of 3 chest tubes for the pneumothoraxes, two on the right and one on the left. He also required continuous renal replacement therapy for acute kidney injury likely secondary to acute tubular necrosis in the setting of cardiac arrest. Renal function improved, and the dialysis catheter was removed the next day. He was started on a paralytic vecuronium in the setting of bronchospasm from status asthmaticus. Ketamine, propofol, and fentanyl infusions were continued for sedation. He continued treatment with continuous albuterol, corticosteroids, and azithromycin. Due to concern for left sided atelectasis, bronchoscopy was performed. There was growth of MRSA and H.influenzae on bronchoalveolar lavage, and he completed a course of ceftriaxone and linezolid. On the 5th day of being intubated, he received modafinil 50 mg to improve his mental status and continued somnolence despite being off sedation for the last day. He became more alert, could localize to voice, and track with his eyes. His RASS score changed from −4 (deep sedation) to 0 (alert and calm). After being on mechanical ventilation for 6 days, somnolence greatly improved, and he passed a spontaneous breathing trial (SBT) and was extubated. The next day, he required re-intubation for worsening hypoxia. CXR showed worsening of the left pneumothorax. The chest tube was taken off water seal and placed back on suction. He was extubated again in 2 days and remained off mechanical ventilation. Chest tubes were removed after being in place for 15 days. He was seen by speech therapy and cleared for regular diet and thin liquids. He was seen by nutrition for protein and calorie loss malnutrition. He also received physical therapy. He was discharged to an inpatient rehabilitation facility. He was discharged without modafinil as he only required one dose the day prior to his first extubation.

## 4 Discussion

Methamphetamine dependence is a destructive illness with half of patients also having a current or past psychiatric disorder. People who use methamphetamine are more likely to be women and have a higher rate of drug injection [1]. Methamphetamine can be administered by intranasal sniffing, pulmonary inhalation, injection, or oral ingestion. It is available in pure crystalline hydrochloride salt or formulated tablets. The effects include euphoria which can last much longer than with the use of cocaine [4]. Methamphetamine and modafinil are both psychostimulants, but they have different effects in the long-term on cognition. Methamphetamine, which is a schedule II substance per the drug enforcement administration (DEA) is addictive and can lead to cognitive decline. In contrast, modafinil is not addictive and can enhance cognition. For example, in a study by Gonzalez et al., 2018 they found that methamphetamine decreased total histone 3 and 4 acetylation and increased total DNA methylation in the prefrontal cortex. In comparison, modafinil increased histone 3 acetylation and mRNA levels of alpha(1B) adrenoreceptor in the prefrontal cortex [5]. This study suggests that use of methamphetamines impairs memory, while use of modafinil does not show cognitive impairment when these epigenetic factors are considered. There is no approved medical therapy for methamphetamine dependency, but there has been promise in the use of modafinil as a medication that can promote wakefulness with little adverse effects [6].

Modafinil has stimulant-like activity, which could decrease the symptoms of amphetamine withdrawal. Hypersomnolence and fatigue are the most prominent features of methamphetamine withdrawal syndrome. For instance, in a study by McGregor et al., they found that people who received modafinil felt more active, alert, and energetic. They also felt less agitation, anxiety, irritability, anhedonia, and suicidal ideation [7]. Studies like this one need to be expanded to include mechanically ventilated patients with history of methamphetamine use who are experiencing methamphetamine withdrawal syndrome. Both of our patients had a history of methamphetamine use and were somnolent during SBT. This hypersomnolence did not allow for safe extubation in either case. Our first patient responded to a 200 mg dose of modafinil, while our second patient responded to a 50 mg dose of modafinil. In a study by Anderson et al., participants were randomly assigned 400 mg modafinil, 200 mg modafinil, or matched placebo daily for 12 weeks with a follow up assessment 4 weeks after treatment. This study found that modafinil with group behavioral therapy was not effective in decreasing methamphetamine use but was also likely inconclusive due to inadequate medication compliance [1]. The dose of modafinil needed to treat methamphetamine use and withdrawal symptoms is unclear based on current data. There needs to be further investigation into the dosing of modafinil for the treatment of methamphetamine use and withdrawal.

There have been clinical studies of the effect of methamphetamine use and withdrawal on sleep. Methamphetamine withdrawal has been shown to extend the latency of sleep and increase the total nighttime and daytime sleep. Further studies have also shown that there has been poor night sleep quality with enhanced daytime sleepiness [8]. This effect of methamphetamine on sleep can be extended to the population of mechanically ventilated patients who are undergoing SBT. The effect of poor sleep quality on our mechanically ventilated patients could have certainly contributed to a prolonged time on mechanical ventilation. Our first patient was on mechanical ventilation for 3 days and then 8 days after her second intubation. Our second patient was on mechanical ventilation for 6 days and then an additional 2 days after he required re-intubation for hypoxia.

Sedation in mechanically ventilated patients with history of methamphetamine use can be particularly challenging. The clinician must find the balance between avoiding withdrawal symptoms of hypersomnolence and in contrast severe agitation that can lead to self-extubation. In our first patient, she was sedated with propofol and fentanyl while our second patient required sedation with ketamine, propofol, and fentanyl infusions. A retrospective single-center study by Kram et al. evaluated patients who were mechanically ventilated for greater than 24 hours. They evaluated daily dose of opioid received in patients who had a UDS positive for cocaine and/or amphetamines and those who were stimulant negative. They found no difference in the daily dose of opioid in those with a positive UDS compared to those with a negative one, which could imply that increasing sedation in those with positive UDS may not be necessary [9].

The effect of modafinil on respiratory failure must also be considered. For instance, Parnell et al. found that modafinil had beneficial effects on blood gases and chronic obstructive pulmonary disease exacerbation admissions in a small group of patients with hypercapnic respiratory failure who were not willing to carry out non-invasive ventilation [3]. This small study shows that further research into the respiratory effects of modafinil on mechanically ventilated patients may be warranted.

Both patients had complicated clinical courses that could have certainly contributed to changes in wakefulness, especially with the different duration and dosage of modafinil used. The first patient had a history of opiate withdrawal, subsequent anoxic brain injury, and possible NMS or malignant catatonia. The second patient had a cardiac arrest, severe asthma exacerbation, and potential post-cardiac arrest encephalopathy. The case series of only 2 patients with intricate clinical courses cannot definitively attribute the effect of modafinil on methamphetamine withdrawal. However, given the existing literature that points to the benefits of the use of modafinil in methamphetamine withdrawal, these cases raise awareness of this possible effect. There is a critical need for large, controlled systematic studies with standardized measures of wakefulness and methamphetamine withdrawal to be done in mechanically ventilated patients.

## CRediT authorship contribution statement

**Kristina Akopyan:** Writing – review & editing, Writing – original draft. **Anastasia Engeleit:** Writing – review & editing. **Minh Nguyen:** Writing – review & editing. **Adam Austin:** Writing – review & editing, Supervision.

## Institution work was performed

University of Florida.

All authors have seen and approved the manuscript.

## Funding

All authors declare the absence of financial support.

## Declaration of competing interest

The authors declare that they have no known competing financial interests or personal relationships that could have appeared to influence the work reported in this paper.

## Abbreviation List:

ADHD: attention deficit hyperactivity disorder
OSA: obstructive sleep apnea
GABA: γ-aminobutyric acid
GAD: generalized anxiety disorder
PTSD: post-traumatic stress disorder
UDS: urine drug screen
SBTs: spontaneous breathing trials
NMS: neuroleptic malignant syndrome
CPR: cardiopulmonary resuscitation
ROSC: return of spontaneous circulation
RASS: Richmond-Agitation Sedation Scale
